# Functional Morphometric Analysis of the Furcula in Mesozoic Birds

**DOI:** 10.1371/journal.pone.0036664

**Published:** 2012-05-30

**Authors:** Roger A. Close, Emily J. Rayfield

**Affiliations:** 1 School of Geosciences, Monash University, Clayton, Victoria, Australia; 2 School of Earth Sciences, University of Bristol, Bristol, United Kingdom; Monash University, Australia

## Abstract

The furcula displays enormous morphological and structural diversity. Acting as an important origin for flight muscles involved in the downstroke, the form of this element has been shown to vary with flight mode. This study seeks to clarify the strength of this form-function relationship through the use of eigenshape morphometric analysis coupled with recently developed phylogenetic comparative methods (PCMs), including phylogenetic Flexible Discriminant Analysis (pFDA). Additionally, the morphospace derived from the furculae of extant birds is used to shed light on possible flight adaptations of Mesozoic fossil taxa. While broad conclusions of earlier work are supported (U-shaped furculae are associated with soaring, strong anteroposterior curvature with wing-propelled diving), correlations between form and function do not appear to be so clear-cut, likely due to the significantly larger dataset and wider spectrum of flight modes sampled here. Interclavicular angle is an even more powerful discriminator of flight mode than curvature, and is positively correlated with body size. With the exception of the close relatives of modern birds, the ornithuromorphs, Mesozoic taxa tend to occupy unique regions of morphospace, and thus may have either evolved unfamiliar flight styles or have arrived at similar styles through divergent musculoskeletal configurations.

## Introduction

Although the collectorship curve of Mesozoic birds has risen steeply in recent decades [1], comparatively few functional analyses have focused on this group. In the last few years, however, this has begun to be rectified. Several studies have attempted to characterise the locomotor adaptations of Mesozoic birds, notably those using wing-element proportions (‘Brachial Index’: [2]–[8]) and primary feather lengths [9] to reconstruct aerial niches; and those using multivariate skeletal measurements [10], [11] and section moduli of limb bones [12] to reconstruct diving modes. Although no fossil taxa were analysed, Simons [13] and Simons et al. [14] successfully used multivariate measurements of forelimb skeletal morphology and cross-sectional geometry to predict flight mode and diving behaviour in pelecaniform birds. Bell and Chiappe [15] used a multivariate morphometric approach to statistically infer the ecology of Mesozoic birds in a broader sense, including habitat occupation and foraging behaviour. Nevertheless, a common feature of these studies is that several associated elements are necessary to draw functional inferences.

The furcula, a key osteological component of the avian flight complex, appears to be a prime candidate for shedding light on the aerial capabilities of early birds as it is both morphologically correlated with flight behaviour and frequently preserved in the fossil record. Once considered to be unique to birds, this element has now been documented across Theropoda, and is known for many Mesozoic avian taxa [16]. Formed by midline fusion of the clavicles, the furcula is marked by considerable structural diversity (reviewed by Nesbitt et al. [16]), varying widely in terms of interclavicular angle, profile curvature (U- to V-shapes), anteroposterior curvature, and development of the hypocleideum and articular facets or epicleideum; anatomical terminology follows Baumel and Witmer [17].

Several biomechanical functions have been proposed for the furcula. Traditionally, this element was thought to play a static function: acting as a transverse spacer (bracing the pectoral girdle against the forces of flapping flight; [18]) and serving as an important origin for the flight muscles [19]. However, Jenkins et al.'s [20] high-speed X-ray cinematography of the European Starling suggested the likelihood of a more dynamic role by demonstrating that the furcula experienced dramatic deformations during the wingbeat cycle. Spreading laterally during the downstroke due to centrifugal forces and rebounding during the upstroke as a result of elastic recoil and contraction of the sternocoracoideus, the dorsal tips of the starling furcula were found to expand by nearly 50% over resting position. Jenkins et al. [20] hypothesised that the spring-like behaviour of the furcula might represent an energy-saving adaptation to facilitate respiration, aiding inflation and deflation of the interclavicular air sacs (part of the secondary respiratory system) in some species. Goslow et al. [21] took this further, hypothesising that the furcula might store energy to aid in the upstroke. However, Bailey and DeMont [22] experimentally demonstrated that only one of their 17 study species was capable of storing a functionally-significant proportion of the kinetic energy of the wing in their furcula. Nevertheless, as Hui and Ellers [23] noted concerning variation in material properties of the furcula, small changes in elasticity may measurably impact energy usage on long-distance flights, and perhaps Bailey and DeMont were too quick to dismiss the role of kinetic energy storage in the furcula.

More recently, the functional significance of morphological variation in the furcula was investigated by Hui [24]. On the basis of a ‘classical’ morphometric analysis, using ratios of linear measurements to characterise curvature of the clavicular rami, Hui demonstrated that the highly variable morphology of the avian furcula seems to correlate more closely with locomotor function than with phylogeny. Ahistorical discriminant analysis was used to classify individuals from 13 species and 8 orders into ‘soaring’, ‘flapping’, ‘subaqueous’ or ‘partial subaqueous’ categories, achieving a relatively low misclassification rate. On the basis of this modest dataset, Hui concluded that fully subaqueous (‘aquaflying’) fliers are characterised by more V-shaped furculae with strong anteroposterior curvature, while those of soaring birds are most U-shaped with low anteroposterior curvature, and aerial flappers' are more varied, falling somewhere in the middle. These morphological differences were attributed to variation in the muscular configurations of different flight groups, such as the need in wing-propelled diving birds to counter underwater drag with increased thrust, effected by a greater protractive component in the downstroke.

As a single element, often-preserved and with a form that seems to correlate with aerial and aquatic locomotor niches, the furcula would appear to be well suited to elucidating the flight behaviour of fossil taxa. Furthermore, the use of outline analysis should allow more sophisticated analysis of furcular shape than the linear measurements used by Hui [24]. Here, we employ eigenshape analysis to quantify shape variation in a large sample of extant bird furculae, and phylogenetic comparative methods (PCMs) to analyse functional variation in morphospace occupation. In particular, we make use of Motani and Schmitz's [25] phylogenetic Flexible Discriminant Analysis (pFDA) to predict flight styles in 21 pre-modern avian taxa. Since most Mesozoic bird fossils lack three-dimensional preservation, two-dimensional eigenshape analysis was considered sufficient for our purposes; although morphometric tools for 3D surface or curve analysis exist, collecting data from an adequately large sample of Mesozoic specimens would be problematic. However, a hybrid approach, also tested, in which data from profile and lateral views were analysed together is one approach that can be applied to three-dimensionally-preserved fossil furculae with greater ease.

The aims of this study are twofold: firstly, to rigorously test the morphofunctional correlation proposed by Hui [24] by applying more sophisticated shape analysis and up-to-date phylogenetic comparative methods to a significantly larger and more representative extant dataset; and, secondly, to use this framework to shed light on the flight behaviour of pre-modern Mesozoic birds such as ornithurines, enantiornithines and more basal taxa.

## Materials and Methods

### Institutional Abbreviations

**AMNH**, American Museum of Natural History, New York; **CAGM**, Chinese Academy of Geological Sciences, Beijing; **DNHM**, Dalian Museum of Natural History, Dalian; **IVPP**, Institute of Vertebrate Paleontology and Paleoanthropology, Beijing; **LH**, Las Hoyas Collection, Museo de Cuenca, Cuenca; **LPM**, Liaoning Paleontological Museum, Liaoning; **MCZ**, Museum of Comparative Zoology, Harvard University, Cambridge; **MIG**, Mongolian Institute of Geology; **MOR**, Museum of the Rockies; **MV**, Museum Victoria, Melbourne; **NIGP**, Nanjing Institute of Geology and Paleontology, Chinese Academy of Sciences, Nanjing; **UMNH**, Utah Museum of Natural History; **YPM**, Yale Peabody Museum, New Haven.

### Taxonomic Dataset

Furculae from 87 extant avian species representing 22 orders and 64 families were used in this study. Of these 87 taxa, 26 were recorded as digital surface-scans by the Aves 3D project (http://www.aves3d.org. Accessed 2011 October 26); 60 were from photographs taken by one of us (R. Close) of the osteological element series collection at Museum Victoria, and 11 were derived from published photographs [16]. For the extinct dataset, furculae belonging to 21 Mesozoic avian taxa and seven non-avian theropods were obtained from figures in the literature, or from photographs personally taken in various institutions. Specimens, and their institutional identification numbers, are listed in Tables 1 and 2. All extant specimens we photographed were dried and fully disarticulated. While we cannot rule out deformation resulting from dessication or other post-excision processes, particularly in small specimens, comparison with recently-excised elements suggests that such deformation is limited in extent; specimens considered for inclusion that showed obvious signs of distortion were omitted from the analysis.

**Table 1:** List of specimens in extant/training dataset.

Taxon	Order	Family	Common name	ID	Flight Mode	No.
*Accipiter fasciatus*	Falconiformes	Accipitridae	Brown Goshawk	MV W6645	FG	1
*Aechmophorus occidentalis*	Podicipediformes	Podicipedidae	Western Grebe	YPM 104291	CF	2
*Ajaja ajaja*	Ciconiiformes	Threskiornithidae	Roseate Spoonbill	YPM 102558	FG	3
*Anas platyrhynchos*	Anseriformes	Anatidae	Mallard	AMNH 5847	CF	4
*Anhinga novaehollandiae*	Pelecaniformes	Anhingidae	Australasian Darter	MV B8674	SS	5
*Anhinga rufa*	Pelecaniformes	Anhingidae	African Darter	YPM 103994	S	6
*Anodorhynchus hyacinthinus*	Psittaciformes	Psittacidae	Hyacinth Macaw	MCZ 346739	CF	7
*Aptenodytes patagonicus*	Sphenisciformes	Spheniscidae	King Penguin	MCZ 347208	SUB	8
*Ardea pacifica*	Pelecaniformes	Ardeidae	White-necked Heron	MV B6820	CF	9
*Ardeotis australis*	Gruiformes	Otididae	Australian Bustard	MV B8566	PF	10
*Argusianus agrus*	Galliformes	Phasianidae	Great Argus	AMNH 4969	CF	11
*Cacatua sanguinea*	Psittaciformes	Cacatuidae	Little Corella	MV W5474	CF	12
*Cerorhinca monocerata*	Charadriiformes	Alcidae	Rhinoceros Auklet	MV B12388	CF	13
*Chauna torquata*	Anseriformes	Anhimidae	Southern Screamer	AMNH 3616	CF	14
*Chionis minor*	Charadriiformes	Chionididae	Black-faced Sheathbill	MV W3457	CF	15
*Circus cyaneus*	Falconiformes	Accipitridae	Hen Harrier	MCZ 342125	S	16
*Cochlearius cochlearius*	Pelecaniformes	Ardeidae	Boat-billed Heron	AMNH 3494	CF	17
*Colaptes auratus cafer*	Piciformes	Picidae	Red-shafted Flicker	MV B12384	CF	18
*Colluricincla harmonica*	Passeriformes	Muscicapidae	Grey Shrikethrush	MV B12031	IB	19
*Coracina novaehollandiae*	Passeriformes	Campephagidae	Black-faced Cuckoo-shrike	MV B10770	IB	20
*Corcorax melanoramphos*	Passeriformes	Corcoracidae	White-winged Chough	MV B11506	IB	21
*Corvus coronoides*	Passeriformes	Corvidae	Australian Raven	MV R7711	FG	22
*Corvus mellori*	Passeriformes	Corvidae	Little Raven	MV B10351	FG	23
*Corvus ossifragus*	Passeriformes	Corvidae	Fish Crow	AMNH 1050	FG	24
*Coturnix pectoralis*	Galliformes	Phasianidae	Stubble Quail	MV B9799	PF	25
*Crypturellus cinnamomeus*	Tinamiformes	Tinamidae	Thicket Tinamou	MV B4785	PF	26
*Cuculus canorus*	Cuculiformes	Cuculidae	Common Cuckoo	YPM 105038	CF	27
*Cygnus olor*	Anseriformes	Anatidae	Mute Swan	MCZ 347051	CF	28
*Dacelo novaeguineae*	Coraciiformes	Halcyonidae	Laughing Kookaburra	MV B12052	CF	29
*Diomedea epomophora*	Procellariiformes	Diomedeidae	Southern Royal Albatross	AMNH 1437	S	30
*Diomedea immutabilis*	Procellariiformes	Diomedeidae	Laysan Albatross	MCZ 343050	S	31
*Esacus giganteus*	Charadriiformes	Burhinidae	Beach Stone-curlew	MV B6587	CF	32
*Eudyptes chryosolophus*	Sphenisciformes	Spheniscidae	Macaroni Penguin	YPM 102975	SUB	33
*Eudyptes chrysocome*	Sphenisciformes	Spheniscidae	Western Rockhopper Penguin	MCZ 346428	SUB	34
*Eurostopodus mystacalis*	Caprimulgiformes	Caprimulgidae	White-throated Nightjar	MV W6663	FG	35
*Falco peregrinus*	Falconiformes	Falconidae	Peregrine Falcon	MV W3765	FG	36
*Falco rusticolus*	Falconiformes	Falconidae	Gyrfalcon	MCZ 343335	FG	37
*Fulica atra*	Gruiformes	Rallidae	Eurasian Coot	MV W6361	CF	38
*Geranospiza caerulescens*	Falconiformes	Accipitridae	Crane Hawk	MCZ 343032	S	39
*Grallina cyanoleuca*	Passeriformes	Grallinidae	Magpie-lark	MV B11122	IB	40
*Guttera plumifera*	Galliformes	Numididae	Plumed Guineafowl	AMNH 6415	PF	41
*Gymnorhina tibicen*	Passeriformes	Cracticidae	Australian Magpie	MV B6540	FG	42
*Herpetotheres cachinnans*	Falconiformes	Falconidae	Laughing Falcon	MCZ 337109	FG	43
*Hirundapus caudacutus*	Apodiformes	Apodidae	White-throated Needletail	MV B11129	S	44
*Larus novaehollandiae*	Charadriiformes	Lariidae	Silver Gull	MV W6163	FG	45
*Leipoa ocellata*	Galliformes	Megapodiidae	Malleefowl	MV B9276	PF	46
*Leptoptilos dubius*	Ciconiiformes	Ciconiidae	Greater Adjutant	MV W5083	S	47
*Limosa lapponica*	Charadriiformes	Scolopacidae	Bar-tailed Godwit	MV W4133	CF	48
*Macrocephalon maleo*	Galliformes	Megapodidae	Maleo	MCZ 340355	PF	49
*Megaceryle torquata*	Coraciiformes	Cerylidae	Ringed Kingfisher	YPM 109939	FG	50
*Menura novaehollandiae*	Passeriformes	Menuridae	Superb Lyrebird	MV B12391	PF	51
*Momotus momota*	Coraciiformes	Momotidae	Blue-crowned Motmot	MV 31795	CF	52
*Morus bassanus*	Pelecaniformes	Sulidae	Northern Gannet	MCZ 347043	S	53
*Morus serrator*	Pelecaniformes	Sulidae	Australasian Gannet	MV W4734	S	54
*Mycteria americana*	Ciconiiformes	Ciconiidae	Wood Stork	AMNH 3768	S	55
*Ninox novaeseelandiae*	Strigiformes	Strigidae	Southern Boobook	MV B11547	FG	56
*Numenius arquata*	Charadriiformes	Scolopacidae	Eurasian Curlew	YPM 111466	CF	57
*Numida meleagris*	Galliformes	Numididae	Helmeted Guineafowl	MV W6355	PF	58
*Oriolus sagittatus*	Passeriformes	Oriolidae	Olive-backed Oriole	MV B8562	IB	59
*Oxyura australis*	Anseriformes	Anatidae	Blue-billed Duck	MV B5145	CF	60
*Pagodroma nivea*	Procellariiformes	Oceanitidae	Snow Petrel	MV R6590	FG	61
*Pandion haliaetus*	Falconiformes	Accipitridae	Osprey	MCZ 347607	S	62
*Pelecanoides urinatrix*	Procellariiformes	Pelecanoididae	Common Diving-petrel	MV B6759	CF	63
*Phaethon rubricauda*	Phaethontiformes	Phaethontidae	Red-tailed Tropicbird	YPM 110024	FG	64
*Phalacrocorax carbo*	Pelecaniformes	Phalacrocoracidae	Great Cormorant	MV W6577	CF	65
*Phaps elegans*	Columbiformes	Columbidae	Brush Bronzewing	MV B8568	CF	66
*Phoenicopterus ruber*	Phoenicopteriformes	Phoenicopteridae	American Flamingo	MV 8748	CF	67
*Podargus strigoides*	Caprimulgiformes	Podargidae	Tawny Frogmouth	MV B6595	FG	68
*Podiceps cristatus*	Podicipediformes	Podicipedidae	Great Crested Grebe	MV W4196	CF	69
*Pterodroma macroptera*	Procellariiformes	Procellariidae	Great-winged Petrel	MV B10118	S	70
*Ptilonorhynchus violaceus*	Passeriformes	Ptilonorhynchidae	Satin Bowerbird	MV W6490	CF	71
*Pulsatrix perspicellata*	Strigiformes	Strigidae	Spectacled Owl	MCZ 343002	FG	72
*Recurvirostra novaehollandiae*	Charadriiformes	Recurvirostridae	Red-necked Avocet	MV W6194	FG	73
*Rostratula benghalensis*	Charadriiformes	Rostratulidae	Greater Painted Snipe	MV B1196	CF	74
*Rynchops niger*	Charadriiformes	Rynchopidae	Black Skimmer	YPM 107666	CF	75
*Sagittarius serpentarius*	Falconiformes	Sagittariidae	Secretarybird	AMNH 4006	FG	76
*Spheniscus humboldti*	Sphenisciformes	Spheniscidae	Humboldt Penguin	MCZ 347040	SUB	77
*Stercorarius skua*	Charadriiformes	Stercorariidae	Great Skua	MV W6658	CF	78
*Stiltia isabella*	Charadriiformes	Glareolidae	Australian Pratincole	MV B8534	CF	79
*Sturnus vulgaris*	Passeriformes	Sturnidae	European Starling	MV B12039	IB	80
*Thalassarche chrysostoma*	Procellariiformes	Diomedeida	Grey-headed Albatross	MV B6731	S	81
*Threskiornis spinicollis*	Ciconiiformes	Plataleidae	Straw-necked Ibis	MV W3973	FG	82
*Tinamus major*	Tinamiformes	Tinamidae	Great Tinamou	MCZ 342774	PF	83
*Tityra semifasciata*	Passeriformes	Cotingidae	Masked Tityra	MV B10711	IB	84
*Tyrannus melancholicus*	Passeriformes	Tyrannidae	Tropical Kingbird	MV B10637	IB	85
*Tyto alba*	Strigiformes	Tytonidae	Barn Owl	MV B11415	FG	86
*Vanellus miles*	Charadriiformes	Charadriidae	Masked Lapwing	MV W1350	CF	87

Flight mode abbreviations: Continuous Flapping (CF); Flap-Gliding (FG); Intermittent Bounding (IB); Soaring (S); Poor Flight (PF); Subaqueous (SUB). MV specimens were photographed by R. Close; YPM and MCZ specimens were digitised by the Aves 3D Project; and AMNH specimens were taken from photographs published by Nesbitt et al. (2009).

**Table 2:** Mesozoic birds and non-avian theropods used in this study.

Genus	Clade	Age	Institutional ID	Source	No.
*Archaeopteryx*	‘Basal Aves’	Late Jurassic	BMNH 37001	[78]	88
*Cathayornis*	Enantiornithes	Early Cretaceous	IVPP V9769	[79]	89
*Concornis*	Enantiornithes	Early Cretaceous	LH 2814	[80]	91
*Confuciusornis*	‘Basal Aves’	Early Cretaceous	GMV 2133	[81]	92
*Eoalulavis*	Enantiornithes	Early Cretaceous	LH 13500a	[82]	93
*Eoconfuciusornis*	‘Basal Aves’	Early Cretaceous	IVPP V11977	[83]	94
*Hongshanornis*	Ornithuromorpha	Early Cretaceous	IVPP V14533	[84]	95
*Iberomesornis*	Enantiornithes	Early Cretaceous	LH 22	[85]	96
*Longicrusavis*	Ornithuromorpha	Early Cretaceous	PKUP V1069	[86]	97
*Longipteryx*	Enantiornithes	Early Cretaceous	IVPP V12325	[87]	98
*Noguerornis*	Enantiornithes	Early Cretaceous	LP 715 IEI	[88]	99
Ornithuromorpha *gen et sp. indet.*	Ornithuromorpha	Early Cretaceous	FRDC-05-CM-021	[89]	100
*Pengornis*	Enantiornithes	Early Cretaceous	IVPP V15336	[89]	101
*Protopteryx*	Enantiornithes	Early Cretaceous	IVPP V11665	[90]	102
*Rapaxavis*	Enantiornithes	Early Cretaceous	DNHM D2522	[91]	103
*Sapeornis*	‘Basal Aves’	Early Cretaceous	IVPP V13276	[92]	104
*Vescornis*	Enantiornithes	Early Cretaceous	NIGP 130722	[93]	105
*Zhongjianornis*	‘Basal Aves’	Early Cretaceous	IVPP V15900	[94]	106
*Gansus*	Ornithuromorpha	Early Cretaceous	CAGM CM003	[95]	108
*Anchiornis*	Paraves	Early Cretaceous	LPM B00169	[96]	109
*Bambiraptor*	Dromaeosauridae	Late Cretaceous	AMNH FR30554	[97]	110
*Falcarius*	Therizinosauria	Early Cretaceous	UMNH-VP 14671	[16]	111
*Neimongosaurus*	Therizonosauria	Late Cretaceous	LH V0001	[16]	112
*Oviraptor*	Oviraptoridae	Late Cretaceous	AMNH FR 6517	[16]	113
*Tyrannosaurus*	Tyrannosauridae	Late Cretaceous	MOR 1125	[16]	114
*Velociraptor*	Dromaeosauridae	Late Cretaceous	IGM 100/976	[16]	115

### Flight mode categories

The extant dataset encompasses a diverse range of locomotory behaviours. In order to explore the relationship between form and function in the furcula, and to draw parallels between extant and Mesozoic taxa, it was necessary to quantify this behavioural variation. There are many ways to gauge flight performance: through agility, manoeuvrability, speed and efficiency, to name but a few. However, we elected to use flight style or ‘mode’ as it is most broadly informative about a species' flight behaviour. Flight mode refers to the style habitually employed during steady, level flight, and does not encompass dynamic aerial behaviours (such as takeoff, landing, facultative gliding, or general manoeuvring). Unfortunately, though, flight mode is difficult to define quantitatively and classification schemes are essentially qualitative.

Prior studies to utilise flight mode categories have devised schemes governed by their own specific aims—e.g., to predict wingbeat frequencies from morphological or physiological parameters [26]–[29], or to examine broader links between morphology and flight behaviour [13], [14], [24], [30]. The scheme used here draws on several of these.

Pennycuick [27] (but see also [26], [31]) recognises four basic flight styles, including three distinct flapping modes: continuous flapping; two intermittent flapping styles, flap-gliding and intermittent bounding; and soaring. Although these modes represent the essential types, other studies have attempted to capture the more nuanced variation that exists in reality: whilst the kinematic features of these flight styles may be clearly-defined, taxa are not necessarily restricted to one style and, unlike their terrestrial counterparts, aerial gaits—and thus flight styles—exist on a continuum. Bruderer et al.'s [29] radar study of avian wingbeat patterns subdivided the basic categories into continuous flapping; static soaring (utilising thermals or updrafts for lift), dynamic soaring (marine birds that exploiting wind-speed differences around waves); flapping & gliding (species that flap continuously, but also glide for lengthy periods); flap-gliding; partial bounding; and intermittent bounding. The earlier study of Viscor and Fuster [30] conflates some categories while appending others: short-flight birds; hovering or stationary fliers; high-frequency flapping fliers; forward flapping; undulating fliers; and gliding or soaring fliers (styles that are kinematically indistinguishable).

Therefore, we choose to recognise five flight mode categories in this study (Table 1): continuous flapping; flap-gliding; intermittent bounding; soaring; and poor or ‘burst-adapted’ fliers, a category to encompass species that are only capable of very short-range flights (e.g., to escape a predator), and cannot maintain steady, level flight for prolonged periods. In the absence of quantitative flight-style data, taxa were classified via observations of motion-picture footage principally derived from the BBC Motion Gallery (www.bbcmotiongallery.com), ARKive (http://www.arkive.org. Accessed 2011 October 26), and the Internet Bird Collection (http://ibc.lynxeds.com. Accessed 2011 October 26), as well as from descriptions in the literature.

Continuous flapping is observed for many clades and body sizes—from ducks to flamingos—though wing loading tends to be high. Flap-gliding and intermittent bounding, although both forms of intermittent flapping flight, differ in terms of wing kinematics: while intermittent bounders fold their wings tightly against the body to streamline themselves during a non-flapping ‘ballistic phase’, flap-gliders, as the name suggests, hold their wings outstretched and glide [27], [31], [33]. Species that utilise intermittent bounding flight are typically small, but it is not unheard of in larger birds, such as woodpeckers or the Australian wattlebirds (e.g., the Red Wattlebird, *Anthochaera carunculata*). The power fraction (proportion of time spent flapping in a flap-glide or flap-bound cycle) may vary from as low as 0.2 to near 1 (continuous flapping). While it has been suggested that intermittent flapping is more energetically efficient [32], [34], Pennycuick [27] favours the view that flap-gliding consumes no less energy than continuous flapping and bounding even more, but allows the flight muscles to work at greater efficiency by operating at near-maximum power output during propulsive phases.

Although a great many birds alternate active flapping flight with unpowered gliding phases, wings held outstretched (“flapping & gliding” in the terminology of Bruderer et al. [29]), soaring birds actively exploit energy in their atmospheric environment (thermals in the case of static soaring, or wave energy in the case of dynamic soaring; [27]). Static and dynamic soarers are marked by different aerodynamic and anatomical adaptations: higher wing-loadings and wing aspect-ratios among dynamic soarers, and lower aspect-ratio wings with low-to-medium wing loadings and slotted wing tips that serve to increase effective aspect-ratio, maximising gliding efficiency while maintaining manoeuvrability (including minimising circling radius) and take-off performance in thermal soarers [27]. Soaring species tend to be on the higher end of the body-mass spectrum, but some smaller birds of prey (such as the Crane Hawk, *Geranospiza caerulescens*, and the Northern Harrier, *Circus cyaneus*), many smaller marine species, and the highly-aerial swifts (which also glide and flap-glide; [35]) also utilise this flight mode.

### Eigenshape Analysis

In contrast to the ratios of linear measurements used by Hui [24] to quantify the three-dimensional shape of the furcula, we adopted two-dimensional eigenshape analysis. A form of outline analysis based on eigendecomposition of pseudolandmark coordinates placed along outlines or curves (reviewed in detail by [36]), eigenshape analysis is superior to linear measurements in a number of ways. Firstly, the length and shape ratios of Hui [24] are not fully independent, and thus contain less information than could be collected for equivalent effort with landmark, semilandmark or outline-based morphometrics [37]. Furthermore, they do not capture the precise nature of furcular curvature (either in profile or lateral aspects), only the magnitude, nor do they capture interclavicular angle. Lastly, since the majority of Mesozoic bird specimens are preserved in 2D, information about their anteroposterior curvature (primarily useful for discriminating wing-propelled divers from foot-propelled or non-divers) has been destroyed.

**Figure 1:**
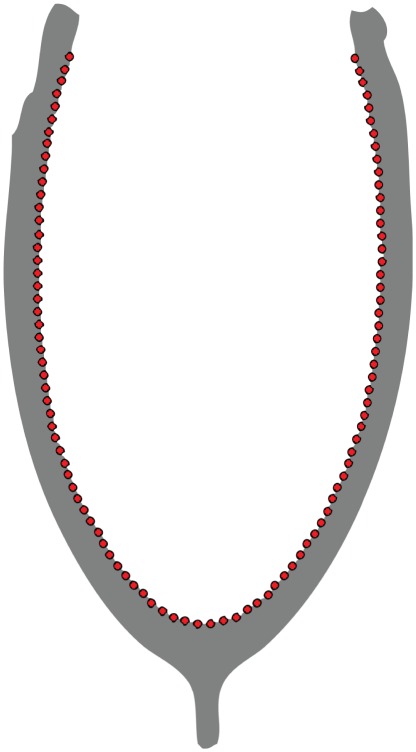
Definition of curves for eigenshape analysis of the furcula in profile view, showing 100 evenly-spaced pseudo-landmark points.

To perform eigenshape analysis, photographs of specimens were first digitised in tpsDig 2.0 (Rohlf 2010). For profile views, furculae were oriented such that the symphysis and junction between the articular omal (epicleideum) regions and clavicular rami lay in the focal plane of the camera. Curves were traced from left to right, encompassing the rami and excluding the epicleideum (Figure 1). We did not wish to include the epicleideum, as it is subject to considerable morphological variation and would complicate the shape analysis, clouding the signals of clavicular curvature considered to be linked to flight adaptations. Furthermore, the region that articulates with the shoulder is less structurally important with respect to muscular attachment and the lateral spreading forces experienced during the downstroke. We found that 100 outline pseudolandmarks, interpolated to achieve equal spacing, captured sufficient morphological detail. Tracing the anterior margin of the clavicular rami in lateral view allowed anteroposterior curvature to be quantified. This relatively crude attempt at capturing three-dimensional shape variation was only tested on the extant dataset, as very few Mesozoic specimens preserve the furcula unflattened.

To determine how clavicular curvature could best be captured in profile view, we ran a morphometric ‘sensitivity analysis’ in which a variety of possible curve-definitions were sampled: the inside curve; the inside curve excluding vestiges of the hypocleideum (an attempt to capture the essential variation in U- to V-shapes as defined by the centroid of the bone); the outside curve including the hypocleideum; full outlines; and both inside and outside curves, with their respective eigenshape scores combined by singular value decomposition (SVD). Extended eigenshape analysis was used for the lateral views to record the extent of curvature in the epicleideum (which can be a significant part of the overall anteroposterior curvature and, unlike in profile view, conveys meaningful functional information); a landmark was placed at the interface between the ramus and the articular area to demarcate the functional division.

Digitised curves were analysed using the Standard and Extended Eigenshape Analysis Mathematica routines written by Jonathan Krieger (Version 2.5; www.morpho-tools.net). Analyses (standard for profile views and extended for lateral views) were conducted using open curves, mean centred, and eigenshape scores produced by SVD using a correlation matrix, as scaling information was not available for all specimens; conversion of the Cartesian (x,y) coordinates to a 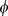-function (taking the net angular deviation between outline coordinates) removed size information, leaving only shape differences. Separate eigenshape analyses were conducted for extant taxa only, for extant and Mesozoic birds, and for extant-plus-extinct birds together with non-avian theropods. This enabled us to first quantify strength of the form-function relationship in extant birds, and to subsequently predict flight modes for extinct taxa. Although separate eigenshape analyses for each combination of taxa may alter the precise nature of the quantified shape variation, its magnitude in each dataset is maximised.

### Phylogenetic Comparative Methods

It is widely recognised that the interrelatedness of data points in biological datasets violates assumptions of traditional statistical methods [38]–[41] and can lead to elevated Type I errors [42]. For this reason, phylogenetic comparative methods were favoured over ahistorical tests. All statistical analyses were conducted in R 2.13.1 (CRAN Project, [43]. R FAQ. Available: http://cran.r-project.org/doc/FAQ/R-FAQ.html. Accessed 13 April 2012) using the ape [44], geiger [45] (CRAN - Package geiger. Available: http://cran.r-project.org/web/packages/geiger/index.html. Accessed 13 April 2012), picante [46], phytools [47] (CRAN - Package phytools. Available: http://CRAN.R-project.org/package=phytools. Accessed 13 April 2012) and adephylo [48] packages.

#### Composite Phylogeny

**Figure 2:**
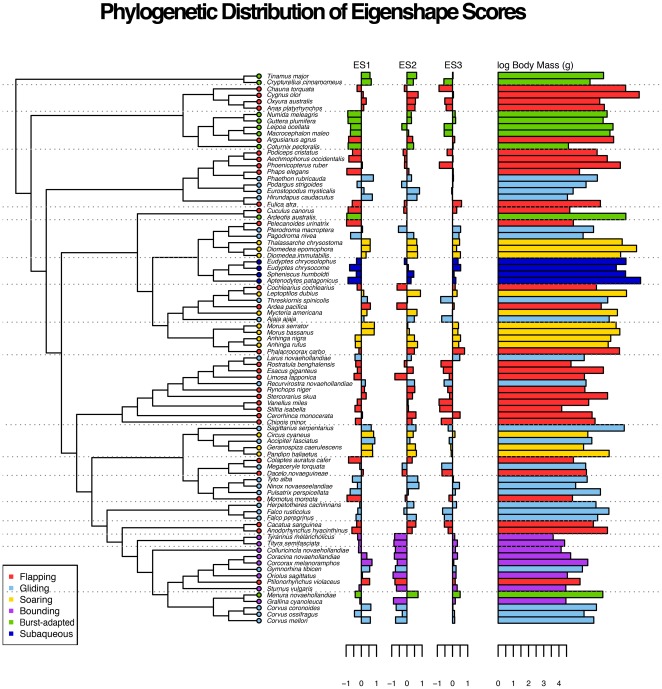
Eigenshape scores and log-transformed body mass data, coloured by flight mode, plotted adjacent to the composite phylogeny (scaled arbitrarily for ease of visualisation) for the extant taxa, allowing visualisation of the phylogenetic signal in flight mode and furcular morphology.

A composite phylogenetic tree (Figure 2) for use with PCMs was constructed in Mesquite 2.75 [49] (Mesquite. Available: http://mesquiteproject.org. Accessed 13 April 2012). The topology was based at an ordinal level on the mitochondrial study of Hackett et al. [50], which has recently received support from the retroposon analysis of Suh et al. [51]. Additional phylogenetic studies were consulted to resolve the intra-ordinal relationships not sampled by [50]: Barker et al. [52] for Passeriformes, Livezey [53] for Charadriiformes, and Lerner and Mindell [54] for Falconiformes and Accipitriformes. The topology for our Mesozoic bird dataset was derived from the recent cladistic analysis of O'Connor et al. [55], while non-avian theropod relationships follow Turner et al. [56].

Because of the composite nature of the phylogeny, branch lengths could not be obtained directly from the aforementioned studies. Several scaling methods were evaluated, including arbitrary methods such as Grafen's [57] 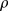 (performed using Manabu Sakamoto's unpublished rho.branch() function) and that of Pagel [39], accomplished using Mesquite 2.54; Blomberg et al.'s [58] Ornstein-Uhlenbeck transform (using the ouTree() function in geiger); and the semi-arbitrary approach of Brusatte et al. [59] that is based on Ruta et al. [60]; applied using Graeme T. LloydÕs R script for dating phylogenetic trees containing fossil taxa: http://graemetlloyd.com/methdpf.html. Accessed 2011 October 26]. In the latter method, branch lengths are shared equally between dates specified for the root and all terminal nodes; internal node ages are not directly derived from phylogenetic analyses.

Ultimately, however, we adopted the more ‘realistic’ approach advocated by Schmitz and Motani [61], in which internal node ages were assigned using a combination of molecular divergence estimates from TimeTree.org [62] for crown-group birds, and dates estimated by O'Connor et al. [55] for Mesozoic lineages. Divergence dates for non-avian theropods were obtained from [63]. Terminal taxon ages for extinct taxa were defined using fossil ranges, and set to 0 Ma for extant taxa. Where divergence dates were not available (e.g., for splits within families or genera), branch-lengths were shared equally. Assignment of node ages and scaling of branches was performed in R using Gene Hunt's scalePhylo() function using a vector of all node and tip ages (available at https://stat.ethz.ch/pipermail/r-sig-phylo/attachments/20110311/5c0c7568/attachment.obj. Accessed 2011 October 26.)

Transformation of branch lengths to conform to Brownian Motion (BM) assumptions was not necessary for either the Phylogenetic Eigenvector Regression, the estimation of Blomberg et al's [58] K (which seek to estimate departure from BM) or the pFDA routine (which corrects for phylogenetic bias). However, as the phylogenetic (M)ANOVA assumes BM character-state evolution, the fitContinuous() function in geiger [64] was used to infer the suitability of this evolutionary model by comparing the second order, or bias-corrected, Akaike Information Criterion (AICc) for a range of fitted models including BM, Ornstein-Uhlenbeck (OU), Early Burst (EB) and white-noise. Because the shape variables were found to depart from BM evolution, branch lengths were transformed using the power.branch() function written by Manabu Sakamoto (pers. comm.) prior to the latter analysis.

#### Detecting Phylogenetic Signal

Several methods were used to detect phylogenetic signal in the morphometric data. Blomberg's K statistic, a measure of phylogenetic autocorrelation developed by Blomberg et al. [58], was implemented via the multiPhylosignal() function in the package ‘picante’ [46]; a value of K>1 corresponds to stronger phylogenetic signal than would be expected for a BM model of character-state evolution, while K<1 indicates a weaker signal. Abouheif's [65] test for serial independence (TFSI), a test for phylogenetic signal equivalent to Moran's I statistic was performed using the abouheif.moran() function in the package ‘adephylo’ [66]. Phylogenetic Eigenvector Regression (PVR; [67]) was also performed with R using the ape and picante packages [44], [46]. Additionally, the phylogenetic flexible discriminant analysis (pFDA) R script provided by Schmitz and Motani [61] estimates Pagel's 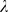, another measure of phylogenetic signal [40] that varies between a value of 0 (no phylogenetic signal) and 1 (strong phylogenetic bias; trait evolution is perfectly described by a BM model).

#### Phylogenetic Analysis of Variance

To test for differences in furcular morphology between locomotor modes, phylogenetic ANOVAs and MANOVAs were performed on eigenshape scores with package ‘geiger’ [64], using 999 iterations to derive the phylogenetic p-value. Eigenshape variables were found to satisfy requirements for multivariate normality and homoscedasticity. A phylogenetic implementation of Tukey's HSD (unpublished R script by Daniel Hanley) was used for post-hoc pairwise comparisons between the flight modes. For comparative purposes, ahistorical variants of these tests were also conducted.

#### Phylogenetic Flexible Discriminant Analysis

**Figure 3:**
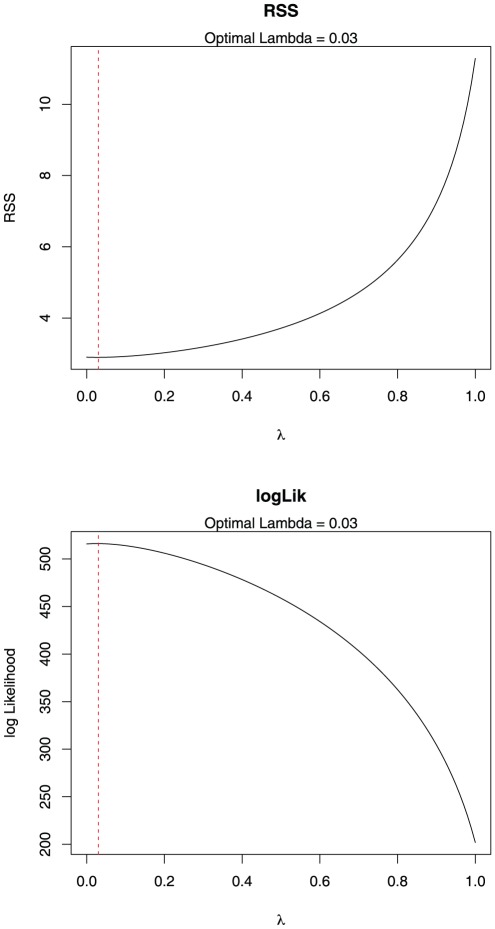
Log-likelihood plots showing optimum value of Pagel's 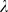 used to control for phylogenetic non-independence in the phylogenetic Flexible Discriminant Analysis (pFDA).

Flight modes of unknown taxa were predicted from furcular morphology using phylogenetic flexible discriminant analysis (pFDA; implemented using the R functions made available by [61]; see also [25]). Phylogenetic flexible discriminant analysis corrects for phylogenetic autocorrelation by combining phylogenetic generalised least squares (PGLS) regression with flexible discriminant analysis, a generalisation of linear discriminant analysis (LDA). The degree of phylogenetic bias removed (assuming BM evolution) can be varied by adjusting the value of Pagel's 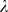 [40]; the appropriate value was found by searching for the 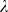 that maximised the log likelihood of the linear fit between the phylogenetically-corrected matrices containing the continuous and categorical data for each specimen [25] (Figure 3).

In addition to the extant flight mode categories listed above, non-avian theropod taxa were scored as a ‘preflight’ locomotor category due to the general morphological similarity between the furculae of land-bound, non-avian theropods and some basal birds. However, this category may be difficult to define: it is far from universally accepted that *Archaeopteryx* was capable of powered flight (e.g., [18]; [68]; [69]), and a recent phylogenetic analysis [70] has even offered weak support for placement within the Deinonychosauria (although this was rapidly refuted by the Maximum Likelihood and Bayesian analysis of Lee and Worthy [71]). Furthermore, non-avian theropods such as *Anchiornis* possess many of the flight-related adaptations of basal birds.

## Results

### Extant-only Dataset

#### Eigenshape Analysis

As expected, the specific aspects of morphological variation captured by the eigenshape analysis differ between the two datasets (extant, and extant-plus-fossil taxa). Visual inspection of morphospace plots and phylogenetic analysis of variance tests determined that the the inside curve, including the hypocleideum, resulted in the greatest inter-group separation for the extant-only dataset, while the outside curve was most successful for the full dataset of extant and extinct taxa.

The first eigenshape, ES1, represents 42.2% of the total variance; ES2, 29.4%; and ES3, 12.1%, collectively accounting for 83.8%. Subsequent eigenshapes account for significantly smaller proportions of the shape variation and appear to have little explanatory significance, much of it corresponding to surface irregularities of the bone or sampling error in the placement of the pseudolandmarks. Only the first three eigenshapes were retained for subsequent analyses, as no significant differences between flight groups were found for scores of less significant eigenshapes, and including them in the pFDA only served to increase misclassification rates.

**Figure 4:**
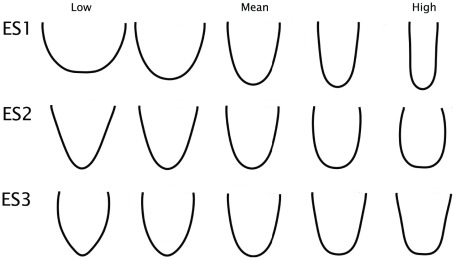
Functionally significant eigenshape models of the furcula in profile view for the extant dataset, produced with the Standard and Extended Eigenshape Analysis Mathematica routines of Jonathan Krieger (Version 2.5).

Models of the first three eigenshapes reveal the predominant axes of shape variation in the furcula (Figure 4). ES1 largely equates with interclavicular angle (low scores representing a large and high scores representing narrow angles), a character traditionally used in cladistic analyses of non-neornithine birds (e.g., [55]). Regressing ES1 against body mass using the phyl.RMA() phylogenetic reduced major axis regression function in phytools reveals it to be weakly but significantly correlated with body mass (Multiple R-squared: 0.1673, Adjusted R-squared: 0.1574; p-value: 9.242e-05). ES2 primarily represents differences in curvature of the clavicular rami, with low scores corresponding to more V-shaped furculae and high scores to U-shapes. ES3 captures the sharpness of the curvature at the symphysis (low values are pointed; high values more rounded), and whether the omal region of the furcula flares medially (low scores) or laterally (high scores).

**Figure 5:**
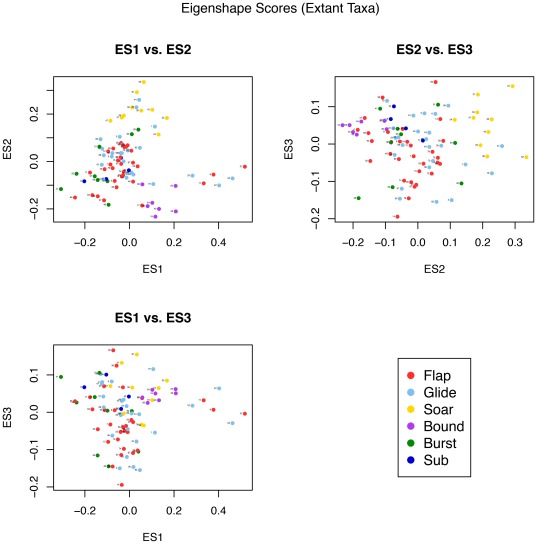
Bivariate morphospace plots of ES1, ES2 and ES3 for the extant-only dataset. Species are identified by numbers listed in Table 1.

Bivariate plots of the first three eigenshapes reveal visual separation of some of the flight groups in morphospace (Figure 5). Soaring and intermittent-bounding taxa are most obviously distinct, separated predominantly along ES2. Soaring birds occupy a distinct region as a result of high ES2 scores and low-to-neutral ES1 scores. Three flap-gliding taxa also plot in this region; two are large species (*Accipiter fasciatus* and *Sagittarius serpentarius*) that might be expected to occasionally encroach on soaring behaviour, while the third, the White-throated Needletail, *Hirundapus caudacutus*, is characterised by very wide, low aspect ratio wings for such a small body size (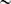
95 g; [72]), reflecting the atypical gliding and soaring capabilities of apodids (in addition to very fast flapping flight; [35]).

On the opposite end of the flight-mode spectrum, intermittent bounders cluster relatively tightly at low values of ES2 and moderate values of ES1. This clustering of intermittent bounders is also apparent in the plots of ES2 vs ES3 and ES1 vs ES3. Flappers and flap-gliders display minimal separation in morphospace, although there is perhaps a slight tendency for flappers to plot at more negative ES2 scores and for flap-gliders to score more positively; such a distribution might be expected given the spectrum-like nature of flight-style niches. Poorly-flighted birds fall in a broadly similar region to flappers and other generalists, while subaqueous fliers plot loosely at moderately positive values of ES3 and moderately negative values of ES2.

**Figure 6:**
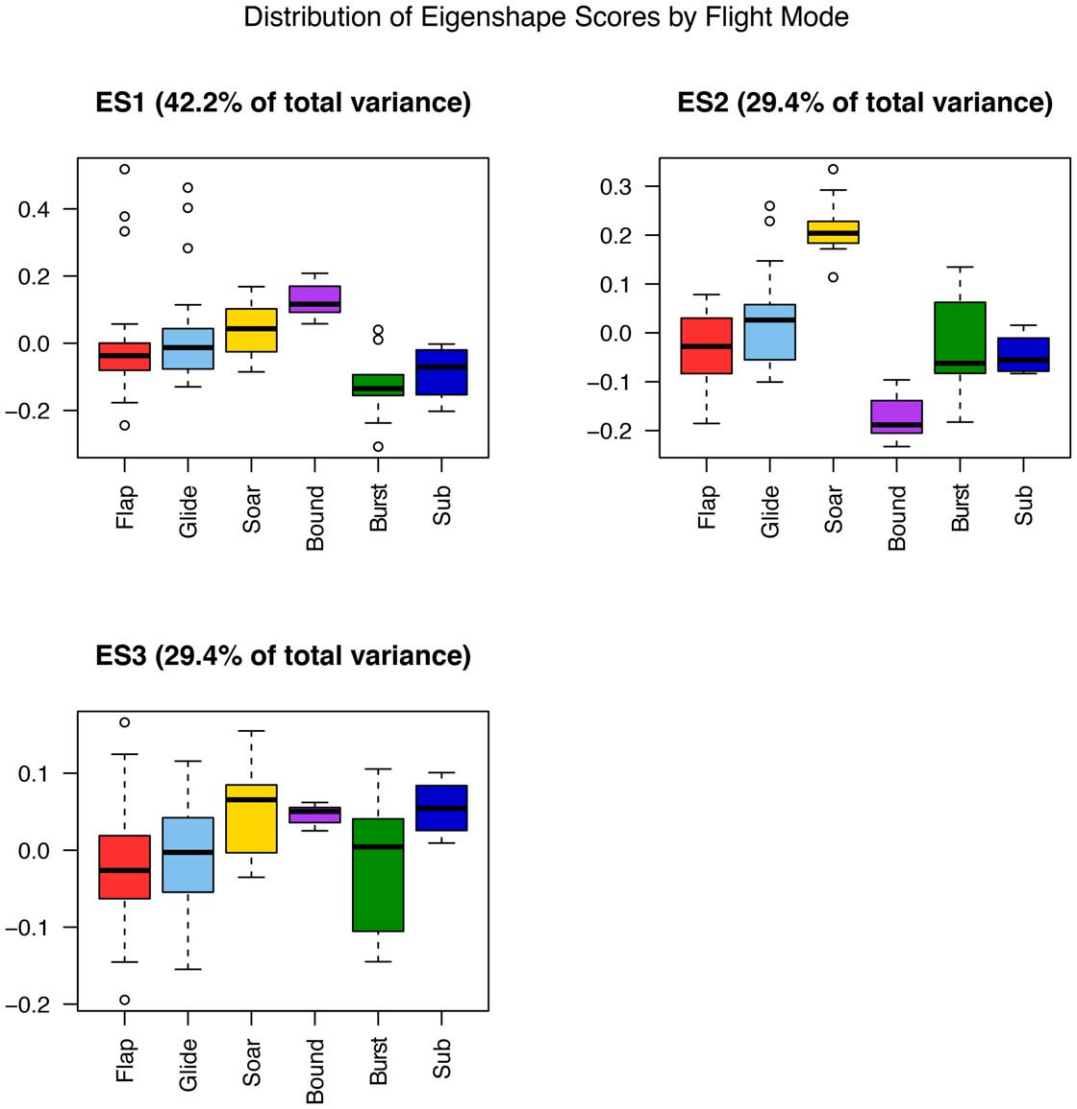
Box-and-whisker plot of eigenshape scores for the extant dataset.

Eigenshape analysis of the curvature of the anterior edge of the furcula in lateral view (Figure 6) reveals fewer differences in morphology between flight groups than profile view: eigenshape scores for flapping, flap-gliding, soaring and bounding taxa are broadly similar on the first eigenshape. However, burst-adapted are marked by strongly negative values of ES1, reflecting an absence of anteroposterior curvature, while non-volant wing-propelled diving birds (i.e., penguins) score very high as a result of their strong anteroposterior curvature. However, certain raptors, notably the diving Osprey *Pandion haliaetus*, also display strong anteroposterior curvature, as do many semi-aquatic taxa not known to engage in wing-propelled diving. Subsequent eigenshapes from the lateral view do not appear to have particular functional significance insofar as flight modes are concerned, although ES2 corresponds to curvature concentrated near the omal tips (low values) or symphysis (high values).

#### Phylogenetic Signal

**Figure 7:**
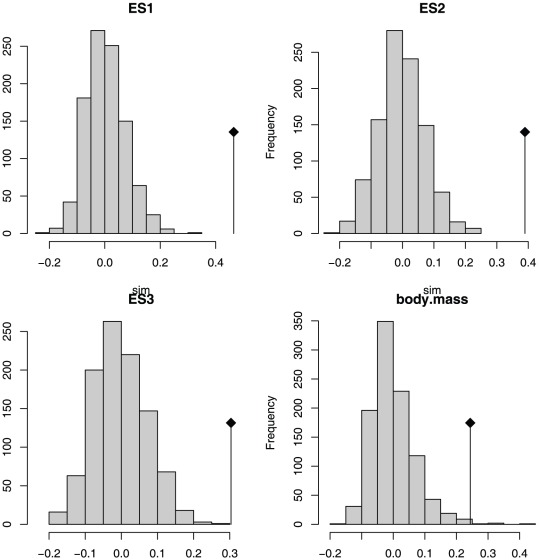
Abouheif's Test for Serial Independence for first three eigenshapes, showing significant phylogenetic signal in all eigenshapes retained.

Blomberg et al.'s [58] test shows low but significant phylogenetic signal in ES1, ES2 and ES3 (results from all tests summarised in Table 3). Abouheif's [65] TFSI also shows significant phylogenetic signal for all of the first three eigenshapes (Figure 7). In contrast, the multivariate PVR of Diniz-Filho et al. [67] was only significant for one of the six phylogenetic eigenvectors (V2) recommended for inclusion by the broken stick model, which together explain 88.03% of the total phylogenetic variance), and overall the regression was not significant (Multiple R-squared: 0.1197, Adjusted R-squared: 0.05367, p-value: 0.107). However, using the Akaike Information Criterion (AIC) step-function improved the model for the first three eigenshapes (Multiple R-squared: 0.09299, Adjusted R-squared: 0.08232, p-value: 0.00408, AIC = −324.45). Additionally, the value of 0.03 estimated for Pagel's 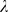 supports a low but significant phylogenetic signal (Figure 3). Plotting eigenshape scores adjacent to the composite phylogeny further highlights the way in which similar furcular morphologies tend to cluster according to clades, particularly at narrower taxonomic levels (Figure 2).

**Table 3:** Results from tests to determine strength of phylogenetic signal in major eigenshapes of extant avian furculae.

Trait	*K*	*P* (Blomberg's *K*)	Observed	*P* (Abouheif's TFSI)	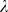	*P* (Pagel's 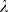 )
ES1	0.02086083	0.529	0.47	<0.001	0.5990652	<0.00005
ES2	0.05194127	0.005	0.39	<0.001	0.8516375	<0.0001
ES3	0.03579084	0.058	0.30	<0.001	0.7546632	<0.001

‘K’ corresponds to strength of phylogenetic signal estimated by Blomberg et al.'s (2003) *K* statistic, and ‘P’ to associated p-value for significance of phylogenetic signal. ‘Observed’ corresponds to the observed ‘C’ statistic from Abouheif's (1999) TSFI. ‘
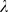
’ corresponds to method of Pagel (1999).

#### Phylogenetic Comparative Methods

For the dataset of extant taxa, a MANOVA of ES1+ES2+ES3 shows significant differences in eigenshape scores between flight groups (*P* = <0.005). However, ANOVAs of individual eigenshapes only find significant differences for the first eigenshape (P = <0.001). This is in contrast to the ahistorical (non-phylogenetic) (M)ANOVAs, which find there to be significant differences for ES1 and ES3 (ES1+ES2+ES3 *P* = 4.485e-12; ES1 *P* = 8.824e-12; ES2 *P* = 0.05415; ES3 *P* = 0.003524); this discrepancy is likely attributable to the inflated Type I error rate common to ahistorical statistical tests applied to interrelated biological datasets. Pairwise comparisons using phylogenetic Tukey's HSD (developed by D. Hanley 2011) find 8 pairwise differences between flight mode groups for ES1+ES2+ES3; 5 for ES1; and two each for ES2 and ES3 (Table 4).

**Table 4:** Pairwise comparisons for MANOVAs of eigenshapes using D. Hanley's phylogenetic implementation of Tukey's HSD.

Post-hoc multiple comparisons for (M)ANOVAs of eigenshapes (extant dataset).							
	Trait	Soaring	Poor Flight	Gliding	Flapping	Subaqueous	Bounding
Soaring	ES1–ES3	-	-	X	X	X	X
	ES1	-	-	X	X	X	X
	ES2	-	-	-	-	X	-
	ES3	-	-	-	-	-	X
Poor Flight	ES1–ES3	-	-	X	X	X	X
	ES1	-	-	-	-	-	-
	ES2	-	-	-	-	X	-
	ES3	-	-	-	-	-	X
Gliding	ES1–ES3	X	X	-	-	-	-
	ES1	X	-	-	-	-	-
	ES2	-	-	-	-	-	-
	ES3	-	-	-	-	-	X
Flapping	ES1–ES3	X	X	-	-	-	-
	ES1	X	-	-	-	-	-
	ES2	X	X	-	-	-	-
	ES3	-	-	-	-	-	-
Subaqueous	ES1–ES3	X	X	-	-	-	-
	ES1	X	X	-	-	-	-
	ES2	-	-	-	-	-	-
	ES3	-	-	-	-	-	-
Bounding	ES1–ES3	X	X	-	-	-	-
	ES1	X	X	-	-	-	-
	ES2	-	-	-	-	-	-
	ES3	X	X	X	-	-	-

### Extant plus Mesozoic dataset

#### Eigenshape Analysis

**Figure 8:**
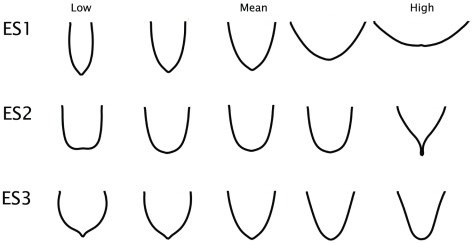
Functionally significant eigenshape models of the furcula in profile view for the full dataset including Mesozoic taxa, produced with the Standard and Extended Eigenshape Analysis Mathematica routines of Jonathan Krieger (Version 2.5).

As the inclusion of extinct birds and non-avian theropods alters the range of morphologies present, eigenshape models for the full dataset represent slightly different aspects of shape variation (Figure 8). For this combination of taxa, the sensitivity analysis recommended the use of the outside curve. Again, only the first three eigenshapes were retained. The first eigenshape explains over half of the sampled morphological variation, and the first three eigenshapes cumulatively account for nearly 90% (ES1: 51.9%; ES2: 21.9%; ES3: 9.4%). Here, ES1 represents interclavicular angle. ES2 and ES3 both capture a combination of the U- to V-shape variation and the degree of development of the hypocleideum as it protrudes or projects dorsally.

**Figure 9:**
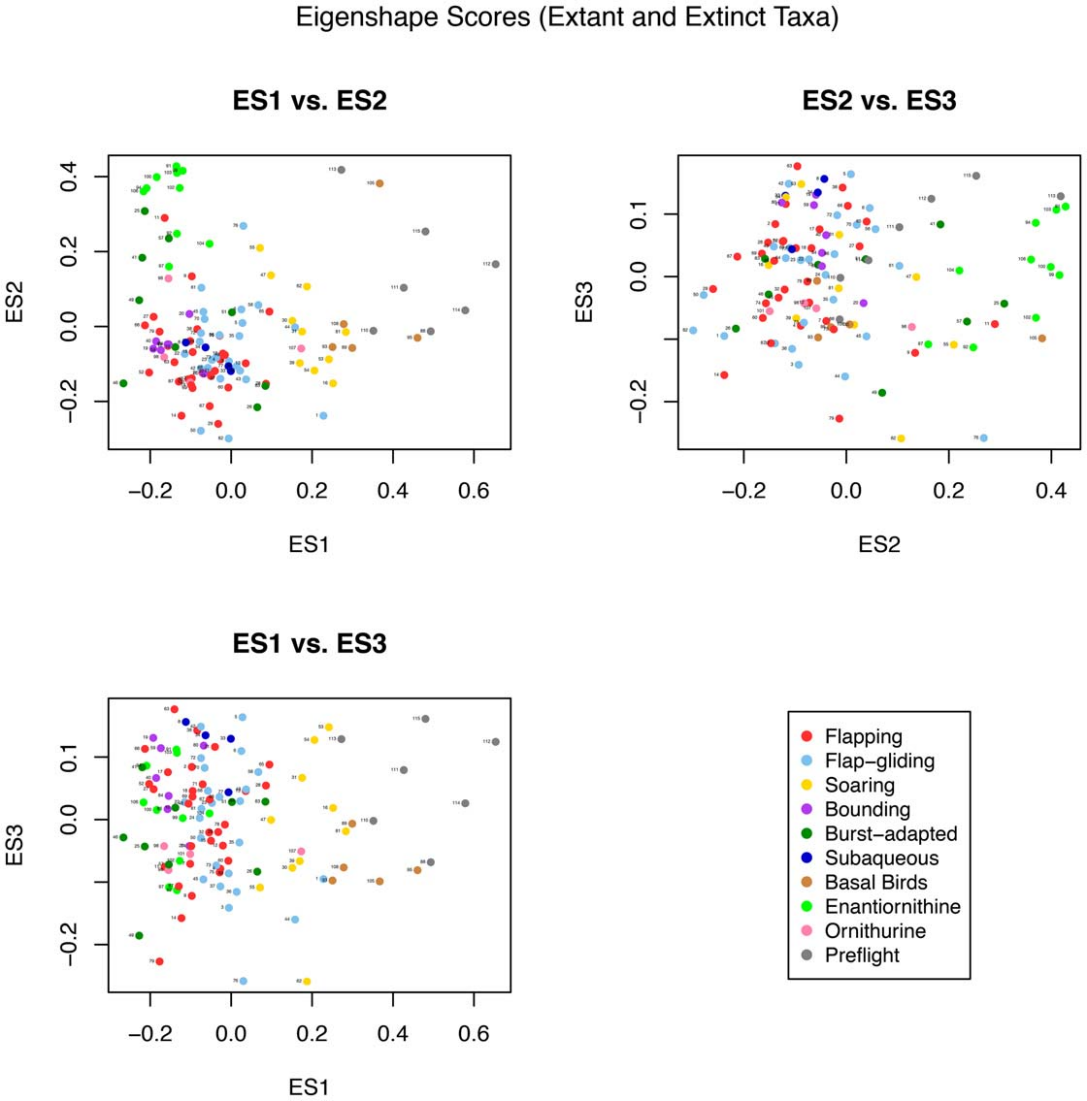
Bivariate morphospace plots of ES1, ES2 and ES3 for the full dataset, including Mesozoic taxa. Species are identified by numbers listed in Tables 1 and 2.

In contrast to the bivariate plot of ES1 vs ES2 for extant taxa, the significant disparity in interclavicular angle between basal birds or non-avian theropods and more derived clades means that no specimens occupy the mean shape (Figure 9). However, this is not the case for ES2 vs. ES3, as these eigenshapes correspond to morphological features not dramatically different between basal and derived birds.

**Figure 10:**
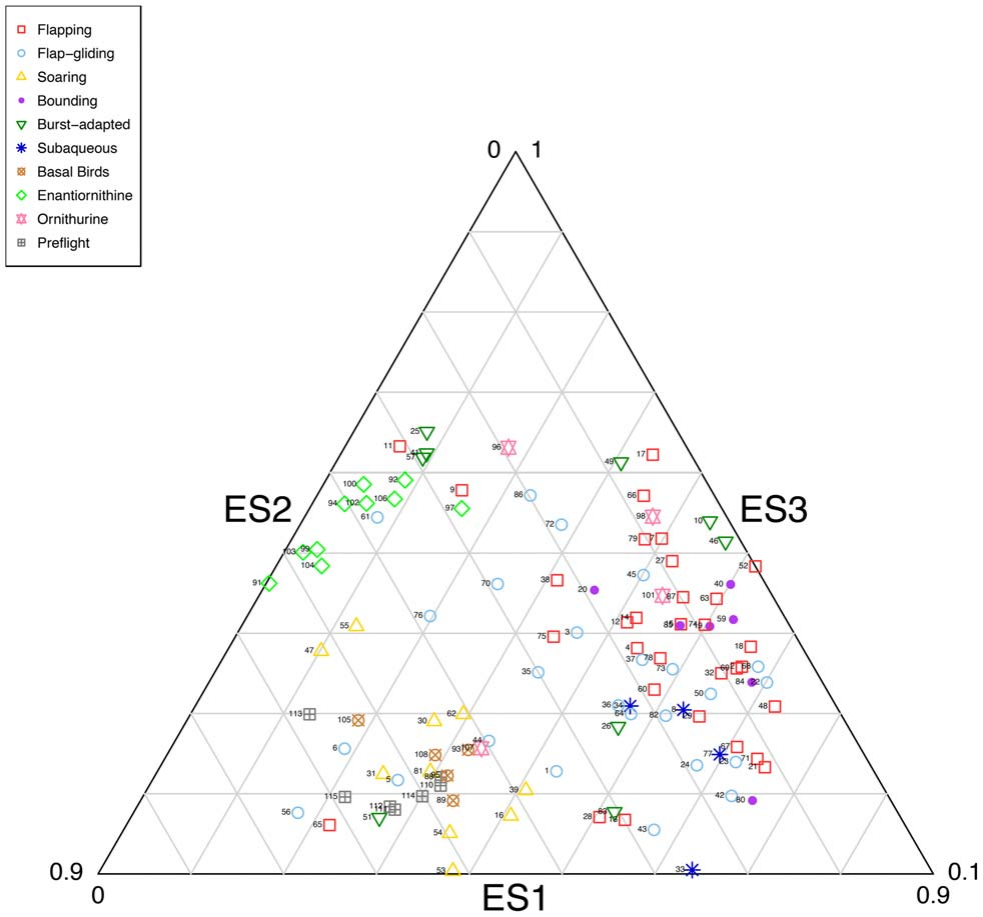
Ternary plot of first three eigenshapes for full dataset, allowing clearer visualisation of the separation of Mesozoic groups in morphospace. Species are identified by numbers listed in Tables 1 and 2.

A ternary diagram representing ES1, ES2 and ES3 (Figure 10) best illustrates the morphospace occupation of extinct and extant taxa. The extremely low ES2 and ES3 scores that characterise enantiornithines set them apart from other clades in morphospace, joined only by a small cluster of burst-adapted birds and very few flapping and flap-gliding taxa. Low scores on ES2 are indicative of V-shaped furculae with straight clavicular rami (regarded as a synapomorphy of Enantiornithes; e.g., [73]), and minimal curvature near the symphysis. Their low ES3 scores reflect an absence of medial curvature near the omal ends of the rami. United by broader interclavicular angles (manifest as high scores on ES1), extant soaring birds are clustered near basal Mesozoic birds and non-avian theropods at the other end of the ternary morphospace. However, basal birds and non-avian theropods tend to occupy a greater extreme than modern soarers, which are somewhat closer to other extant forms. Ornithuromorphs are the only Mesozoic taxa to unequivocally coincide with extant forms in morphospace, intermingling with modern flapping and flap-gliding birds at low values of ES1 and mid-to-high values of ES2. The application of (M)ANOVAs in which Enantiornithes, Ornithurae, basal birds and non-avian theropods are scored as independent factors confirms these apparent differences in furcular morphology (MANOVA of first three eigenshapes significant at *P* = <0.001; for ANOVAs of ES1 and ES2 *P* = <0.001; for ES3 *P* = 0.04). Post-hoc multiple comparisons using phylogenetic Tukey's HSD reveal that ES1 accounts for most significant pairwise differences: ES2 only discriminates non-avian theropods from birds, while ES3 does not discriminate any groups (Table 5).

**Table 5:** Pairwise comparisons for MANOVA of first three eigenshapes using D. Hanley's phylogenetic implementation of Tukey's HSD.

Post-hoc multiple comparisons for ES1-3 (full dataset).											
	Trait	Bound	Poor	Flap	Glide	Sub	Soar	E/ornithine	Basal	Preflight	Ornithurine
**Bounding**	ES1–ES3	-	-	-	-	-	-	-	X	X	X
	ES1	-	-	-	-	-	-	X	X	X	-
	ES2	-	-	-	-	-	-	-	-	X	-
	ES3	-	-	-	-	-	-	-	-	-	-
**Poor**	ES1–ES3	-	-	-	-	-	-	-	X	X	X
	ES1	-	-	-	-	-	-	X	X	X	X
	ES2	-	-	-	-	-	-	-	-	X	-
	ES3	-	-	-	-	-	-	-	-	-	-
**Flap**	ES1–ES3	-	-	-	-	-	-	X	X	X	X
	ES1	-	-	-	-	-	-	X	X	X	-
	ES2	-	-	-	-	-	-	-	-	X	-
	ES3	-	-	-	-	-	-	-	-	-	-
**Glide**	ES1–ES3	-	-	-	-	-	-	-	X	X	X
	ES1	-	-	-	-	-	-	X	X	X	X
	ES2	-	-	-	-	-	-	-	-	X	-
	ES3	-	-	-	-	-	-	-	-	-	-
**Sub**	ES1–ES3	-	-	-	-	-	-	-	-	-	X
	ES1	-	-	-	-	-	-	X	X	X	X
	ES2	-	-	-	-	-	-	-	-	X	-
	ES3	-	-	-	-	-	-	-	-	-	-
**Soaring**	ES1–ES3	X	-	-	X	-	-	-	-	-	X
	ES1	-	-	-	-	-	-	X	X	X	X
	ES2	-	-	-	-	-	-	-	-	X	-
	ES3	-	-	-	-	-	-	-	-	-	-
**E/thine**	ES1–ES3	X	X	X	X	X	-	-	-	-	X
	ES1	X	X	X	X	X	X	-	-	X	X
	ES2	-	-	-	-	-	-	-	-	X	-
	ES3	-	-	-	-	-	-	-	-	-	-
**Basal**	ES1–ES3	X	X	X	X	X	-	-	-	-	X
	ES1	X	X	X	X	X	X	-	-	X	X
	ES2	-	-	-	-	-	-	-	-	X	-
	ES3	-	-	-	-	-	-	-	-	-	-
**Preflight**	ES1–ES3	X	X	X	X	X	X	X	X	X	-
	ES1	X	X	X	X	X	X	X	X	-	X
	ES2	X	X	X	X	X	X	X	X	-	X
	ES3	-	-	-	-	-	-	-	-	-	-
**Ornithurine**	ES1–ES3	-	-	-	-	-	-	X	X	X	X
	ES1	-	X	-	X	X	X	X	X	X	-
	ES2	-	-	-	-	-	-	-	-	X	-
	ES3	-	-	-	-	-	-	-	-	-	-

#### Phylogenetic Flexible Discriminant Analysis

**Figure 11:**
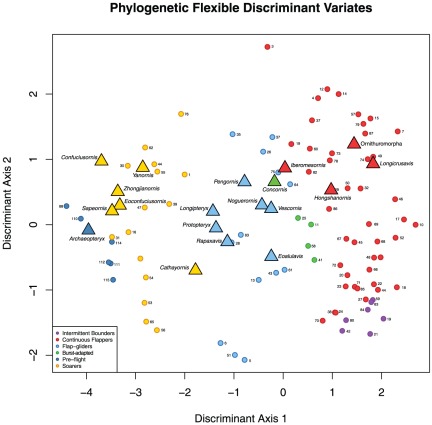
Bivariate plot of discriminant variates using predict function of pFDA. Small circles = training taxa; large triangles = unknown specimens. Colours for both training and unknown samples represent predicted, not predefined, flight modes. Species are identified by numbers listed in Table 1.

**Figure 12:**
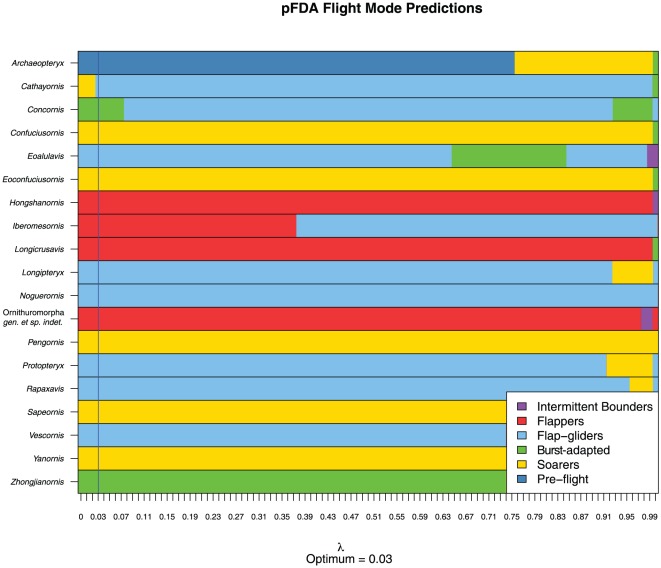
Flight group predictions for Mesozoic taxa using the phylogenetic Flexible Discriminant Analysis of Motani and Schmitz [61]. Flight predictions for optimum value of Pagel's 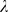 are indicated by the blue vertical line.

Eigenshape scores from the outside curve of the furcula in profile view were found to result in the lowest rate of pFDA misclassifications (Table 6; phylogenetic discriminant variates for training and unknown taxa are rendered in Figure 11). Although the error rate of 0.4 is quite high, this is mainly attributable to the difficulty of discriminating between flapping, flap-gliding and burst-adapted species. More specialised aerial niches are more easily discriminated: 9/10 soaring species are correctly classified; 5/8 intermittent bounding species (the other three being classified as flappers); and 5/7 pre-flight species are correctly classified (with two misclassified as soarers). Predicted flight modes for Mesozoic avian taxa are illustrated in Figure 12, including classifications at other values of 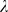. Whilst there is some doubt about the validity of the optimal lambda (a larger sample size would be needed to assure a reliable estimation), classifications generally hold over a wide range of values. Two notable exceptions are *Cathayornis* and *Concornis*, classified as soaring and burst-adapted forms at a 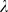 of 0.03, but becoming flap-gliders at slightly higher values. Most ornithurine taxa are classified as flappers, although *Yanornis* is classified as a soarer.

**Table 6:** Cross-classification/confusion matrix from phylogenetic flexible discriminant analysis of full dataset including Mesozoic taxa.

Cross-classification/confusion matrix.						
	Bound	Flap	Glide	Poor	Preflight	Soar
Bound	5	1	1	0	0	0
Flap	3	25	13	3	0	0
Glide	0	3	7	3	0	0
Poor	0	1	0	3	0	0
Preflight	0	0	0	0	5	1
Soar	0	1	4	0	2	9
% Correct	63%	81%	28%	33%	71%	90%

True classifications along top, predicted classifications on left-hand side.

## Discussion

Using modern morphometric and phylogenetic comparative methods, we tested the strength of the apparent correlation between furcular morphology and flight mode in extant avian taxa. Results were then used to predict which of these modern flight modes, if any, best fit species in our Mesozoic dataset. Our findings affirm some earlier conclusions, notably that soaring birds are differentiated from continuously-flapping species by a more U-shaped furcula—although the curvature of the clavicular rami appears to be less consequential than the interclavicular angle, which is unusually broad in soarers and narrow in intermittent bounders. We also confirmed that use of the wings for propulsion underwater is correlated with increased anteroposterior curvature, although furculae of some groups of non-diving birds, notably birds of prey, also exhibit this feature.

The spectrum of avian flight is intricate and varied, and reduction to any set of discrete ‘buckets’ will surely fail to capture every last behavioural adaptation. Myriad selective pressures place contrasting demands on flight capabilities, so trade-offs (between, for example, efficiency and slow-speed manoeuvrability) are inevitable. The steady, level flight on which flight-mode categories are usually based encapsulate only a single aspect of a bird's aerial capabilities: take-off and landing, dynamic, non-steady flight such as hovering, gliding and so on are all functionally important, and may complicate attempts to establish correlations between musculoskeletal design and function if not taken into account. Furthermore, non-aerial locomotory behaviours such as wing-propelled diving may place additional demands and selective pressures on the flight apparatus. However, use of discrete flight categories in this study was necessitated by lack of quantitative methods for characterising flight performance; wing parameters such as aspect ratio, wingtip shape and wing loading may well be useful metrics, but inadequate data has been collected to allow meaningful comparisons. Pending further collection of wing morphology data or quantitative flight mode data, the approach used here remains the best way to characterise flight behaviour.

Of the flight modes analysed for the first time in this study, intermittent bounders were found to be strongly associated with narrow interclavicular angles and straight clavicular rami (their tight clustering in morphospace reflecting limited morphological variation), and short-range or burst-adapted fliers tended to be characterised by minimal anteroposterior curvature, but occupied a broadly similar distribution to flappers in terms of profile-view morphospace. Higher levels of anterposterior curvature in birds of prey may relate to increased thrust requirements stemming from load-carrying behaviour or, in the case of the Osprey, *Pandion haliaetus*, prey-carrying coupled with diving. Flappers and flap-gliders cannot be distinguished by their profile curvature but, together with poor or burst-adapted, are broadly distinct from the more derived flight modes of soaring and intermittent bounding.

The first eigenshape, primarily representing interclavicular angle, confers the greatest degree of separation between flight modes (principally discriminating soaring and intermittent bounders). Regressing ES1 against body mass reveals it to be moderately correlated with body mass, which suggests that it may be related to allometric scaling. However, as Simons et al. [14] have noted, body size or allometric effects are an important aspect of flight adaptations, and it would not be advantageous to remove such effects when the aim is to reconstruct locomotor styles in unknown specimens (by, for example, taking the residuals from a regression). Furthermore, the furcula of the diminutive soaring/flap-gliding White-throated Needletail (*Hirundapus caudacutus*) is sited close to much larger soaring species in morphospace, supporting the notion that flight style, not body size, is the primary influence on furcular morphology.

Unlike species included in the dataset of Hui [24], our taxa were not selected to represent particular extremes of flight behaviour, as this might have skewed predictions for unknown specimens. In combination with our significantly larger dataset and more representative sample of flight modes, this may be responsible for the comparatively poor overall misclassification rate achieved by the pFDA (40% overall, compared to 25% for Hui's ahistorical discriminant analysis; however, much of the error in our study can be ascribed to the nebulous boundary between flapping and flap-gliding species). Accounting for phylogenetic bias may have also increased misclassification rates, although the diminished Type I error rates and higher statistical power of phylogenetic comparative methods nevertheless justify their use. Importantly, though, it is the poor distinction between flappers and flap-gliders that accounts for most of the error; soarers and intermittent bounders are predicted with reasonable precision.

Flight mode predictions for extinct taxa confirm the differences that are apparent in the morphospace plots of raw eigenshape scores. Enantiornithines are markedly distinct from the bulk of modern taxa due to their unusually straight clavicular rami and long hypocleideum (consistent with their characterisation as ‘V-shaped’ in systematic analyses), manifested as very high ES2 scores. Conversely, the more U-shaped ornithurine furculae plot more closely to flappers, while very basal taxa, such as *Archaeopteryx*, *Confuciusornis* and *Sapeornis* plot at even higher values of ES1 than modern soarers (though less extreme than non-avian theropods). As a result, the pFDA struggles to classify the unusually-shaped enantiornithine specimens, often predicting them to be flap-gliders (*Eoalulavis*, *Longipteryx*, *Noguerornis*, *Pengornis*, *Proptopteryx*, *Rapaxavis* and *Vescornis*) or soaring forms (*Cathayornis*)—a highly unlikely outcome given their predominantly small size and visual separation from these flight modes in morphospace. Other enantiornithines are somewhat more plausibly classified, such as the Spanish species *Iberomesornis* (‘flapping’) and *Concornis* (‘burst-adapted’). However, given their comparatively smaller body-sizes (particularly in the Early Cretaceous, though towards the end of the Mesozoic enantiornithines attained much greater proportions; e.g., [74]–[76]) and the numerous flight adaptations apparent elsewhere in their anatomy, intermittent bounding is perhaps more likely for aerodynamic reasons; very few similarly-sized modern species use styles other than flapping or bounding [77]. On the other hand, the general absence of anteroposterior curvature in enantiornithines (unless due to post-depositional flattening) is compatible with poorer powered flight abilities as it may suggest less protraction during the downstroke, and thus poorer thrust generation (accepting Hui's suggestion that anteroposterior curvature is positively associated with a protractive component to the downstroke). The dissimilar furcular morphology of enantiornithines may reflect different muscular configurations to those of modern birds, in much the same way that the greatly elongated hypocleideum has been suggested to have partially taken over the role of an enlarged sternal keel in this group [73].

Although several basal birds are classified as soarers due to their proximity to modern taxa (albeit at greater morphospacial extremes), many morphological differences are not captured by the eigenshape analysis of a single curve (such as their dimensions relative to overall body-size, degree of anteroposterior flattening, and the development of the epicleideum). Their broad interclavicular angles are clearly the product of phylogenetic inertia (between basal-most and more derived birds, the interclavicular angle is seen to narrow markedly), and not primary adaptations for soaring (a derived behaviour in modern birds; [33]). Though fairly robust to different degrees of phylogenetic-bias removal (predictions generally hold for a range of 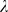 values; Figure 12), sensitivity of the pFDA predictions to variables including branch-length scaling and morphometric curve-selection underscores their unreliability for many pre-modern groups of birds. However, the method shows great promise for informing our understanding of flight in extinct neornithines and more derived species of ornithurine.

Scaling of branch lengths can significantly effect the results of all PCMs used, especially the pFDA. While we opted for scaling based on divergence estimates drawn from analyses presented on TimeTree.org, we also assessed the performance of other commonly-used methods (including those of Brusatte et al. [59]; Grafen [57]; Pagel [39]; Blomberg et al. [58]; and Ruta et al. [60]). Even though we and others (e.g., Schmitz and Motani [61]) consider realistic branch-scaling to be preferable, most PCMs assume BM evolution, so transformation may still be necessary prior to certain analyses. Nevertheless, dramatic differences in phylogenetic discriminant predictions highlight the need for caution when interpreting results from similar studies in which branch lengths are set arbitrarily (if, for example, if all lengths are equal).

Although this study makes use of more sophisticated analytical tools that might be expected to clarify the findings of Hui [24], in fact, a murkier picture seems to emerge. Through a markedly more restricted sample size, careful selection of species displaying unambiguous locomotory adaptations and use of multiple individual measurements as independent data points (in place of species means), Hui's study may have overstated the strength of the form-function relationship in the avian furcula. While we have had some success using furcular morphology to supplement our view of flight in more modern groups of Mesozoic birds, it would appear that the highly unusual situation of enantiornithines and basal-most birds in morphospace limits our ability to infer form from function using this element. However, we demonstrate that eigenshape analysis of the avian furcula allows the more derived flight modes in modern birds to be statistically discriminated in a phylogenetic framework and, as such, these methods should be expected to yield greater success when applied to extinct Tertiary taxa. Additionally, further investigation of form and function in the avian pectoral girdle—be it of the furcula, or other elements, such as the coracoids or sternum—would likely benefit from the marriage of 3D geometric morphometric techniques to phylogenetic comparative methods.
